# Growth of *Plasmodium falciparum* in response to a rotating magnetic field

**DOI:** 10.1186/s12936-018-2333-2

**Published:** 2018-05-03

**Authors:** Rebecca C. Gilson, Robert J. Deissler, Richard F. Bihary, William C. Condit, Mary E. Thompson, D’Arbra Blankenship, Kerry O. Grimberg, Robert W. Brown, Brian T. Grimberg

**Affiliations:** 1 Department of Physics, CWRU College of Arts and Sciences, 2076 Adelbert Road, Cleveland, OH 44106-7079 USA; 2 Department of Pathology, Center for Global Health and Diseases, Biomedical Research Building, Room 427, 2109 Adelbert Road, Cleveland, OH 44106 USA

**Keywords:** Malaria, Hemozoin, Magnetic field

## Abstract

**Background:**

*Plasmodium falciparum* is the deadliest strain of malaria and the mortality rate is increasing because of pathogen drug resistance. Increasing knowledge of the parasite life cycle and mechanism of infection may provide new models for improved treatment paradigms. This study sought to investigate the paramagnetic nature of the parasite’s haemozoin to inhibit parasite viability.

**Results:**

Paramagnetic haemozoin crystals, a byproduct of the parasite’s haemoglobin digestion, interact with a rotating magnetic field, which prevents their complete formation, causing the accumulation of free haem, which is lethal to the parasites. *Plasmodium falciparum* cultures of different stages of intraerythrocytic growth (rings, trophozoites, and schizonts) were exposed to a magnetic field of 0.46 T at frequencies of 0 Hz (static), 1, 5, and 10 Hz for 48 h. The numbers of parasites were counted over the course of one intraerythrocytic life cycle via flow cytometry. At 10 Hz the schizont life stage was most affected by the rotating magnetic fields (p = 0.0075) as compared to a static magnetic field of the same strength. Parasite growth in the presence of a static magnetic field appears to aid parasite growth.

**Conclusions:**

Sequestration of the toxic haem resulting from haemoglobin digestion is key for the parasites’ survival and the focus of almost all existing anti-malarial drugs. Understanding how the parasites create the haemozoin molecule and the disruption of its creation aids in the development of drugs to combat this disease.

## Background

Currently, malaria is the second leading cause of death in the tropical and subtropical regions of the world and, among the five species of malaria, *Plasmodium falciparum* is the deadliest [1]. The parasite matures through three life stages when in human blood: ring, trophozoite, and schizont. At the beginning of the ring stage, the parasite uptakes the cytoplasm of the red blood cells (RBC), called the “Big Gulp” [2]. The haemoglobin is transported to a vacuole where it is digested into peptides that are degraded into amino acids. This process leaves free haem, which consists of an iron atom bound to four nitrogen atoms of the pyrrole ring of protoporphyrin IX with two carboxylic side chains [3]. Free haem is toxic to the parasite because the iron at the centre can alternate between its + 2 and + 3 states, leading to the generation of free radicals, the destabilization of the cell membrane, and the disruption of protein structures [4]. To detoxify the haem, the parasite dimerizes it to form β-haematin through a bond between the iron of the first haem and an oxygen of the carboxylic side chain of the second haem [5]. In addition, β-haematins form hydrogen bonds between the oxygens of their second carboxylic side chains yielding a haemozoin crystal. The haemozoin crystal consists of layers of these long chains, held together by π–π stacking forces [3]. The iron bond in the β-haematin causes the whole haemozoin crystal to be paramagnetic [6–8]. One can, therefore, study the effects of the associated net magnetization of a crystal sample arising from an exposure to an external magnetic field [9–13]. This project sought to exploit the paramagnetic nature of the parasite’s haemozoin to inhibit parasite viability.

## Methods

### Parasite growth

*Plasmodium falciparum* HB3 strain (MR4-155, contributed by T.E. Wellems, NIAID) was obtained from the Malaria Research and Reference Reagent Resource (ATCC, Manassas, Virginia). The parasite cultures were maintained at 4% haematocrit, in RPMI 1640 supplemented with 0.5% Albumax, 80 ng/mL gentamicin, and 8200 ng/mL hypoxanthine, at 37 °C in an incubator containing 5% CO_2_, 1% O_2_, and 94% N_2_ and the media was changed daily [14]. The parasitaemia of the culture was checked daily by microscopy and the parasitaemia was kept under 6%. To synchronize cultures the standard procedures for Plasmagel^®^ were used to collect late stage parasites, and sorbitol, to collect ring-stage parasites [15].

### Magnetic field exposure

**Fig. 1:**
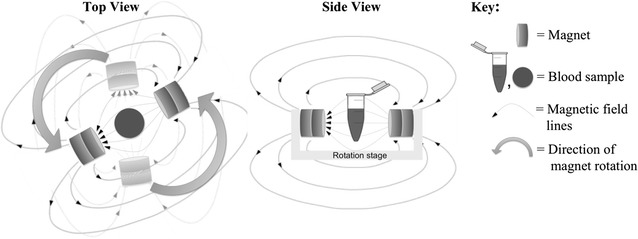
The apparatus consisted of a sample holder in which the test tube remained stationary as the magnets rotated around it. The control samples, not shown, were placed in the same incubator at a location where the magnetic field was negligible

The apparatus utilized two neodymium magnets per sample to produce a rotating 0.46 T magnetic field (Fig. 1), in the centre of the sample. The magnetic field strength was chosen based on previous studies by Newman et al. [16]. Four different cultures were exposed to the magnetic field: rings, trophozoites, schizonts and all three stages mixed together. Two sets of each of these cultures were made, one as a test culture and one as a control culture. Each condition was represented as an average of three replicates. It is important to note that while these cultures started as separate life stages, the parasites in them matured through the whole life cycle and those that survived the treatment returned to their original stage. The magnetic field exposure time of 48 h was chosen because that is the time frame that it takes blood stage malaria parasites to complete 1 full round of maturation. This timeline provided us with the greatest opportunity for us to observe the effects of the magnetic fields on the parasite’s blood stage growth cycle. For example, the parasites in the ring culture started as rings, and during the 48 h matured through the trophozoite and schizont stage to end up as rings again. Eight microtubes containing 450 µL of ~ 1% parasitaemia and 4% haematocrit red blood cells were prepared and gassed with the same mixture in which they were grown. Test samples were exposed to the same magnetic field but at different frequencies of rotation: 0 Hz (static), 1, 5, and 10 Hz. The control samples were placed in the same incubator, but at a distance where the magnetic field was negligible. Flow cytometry was used at these same time marks to reach at least 50,000 cells.

### Data acquisition/flow cytometry

**Fig. 2:**
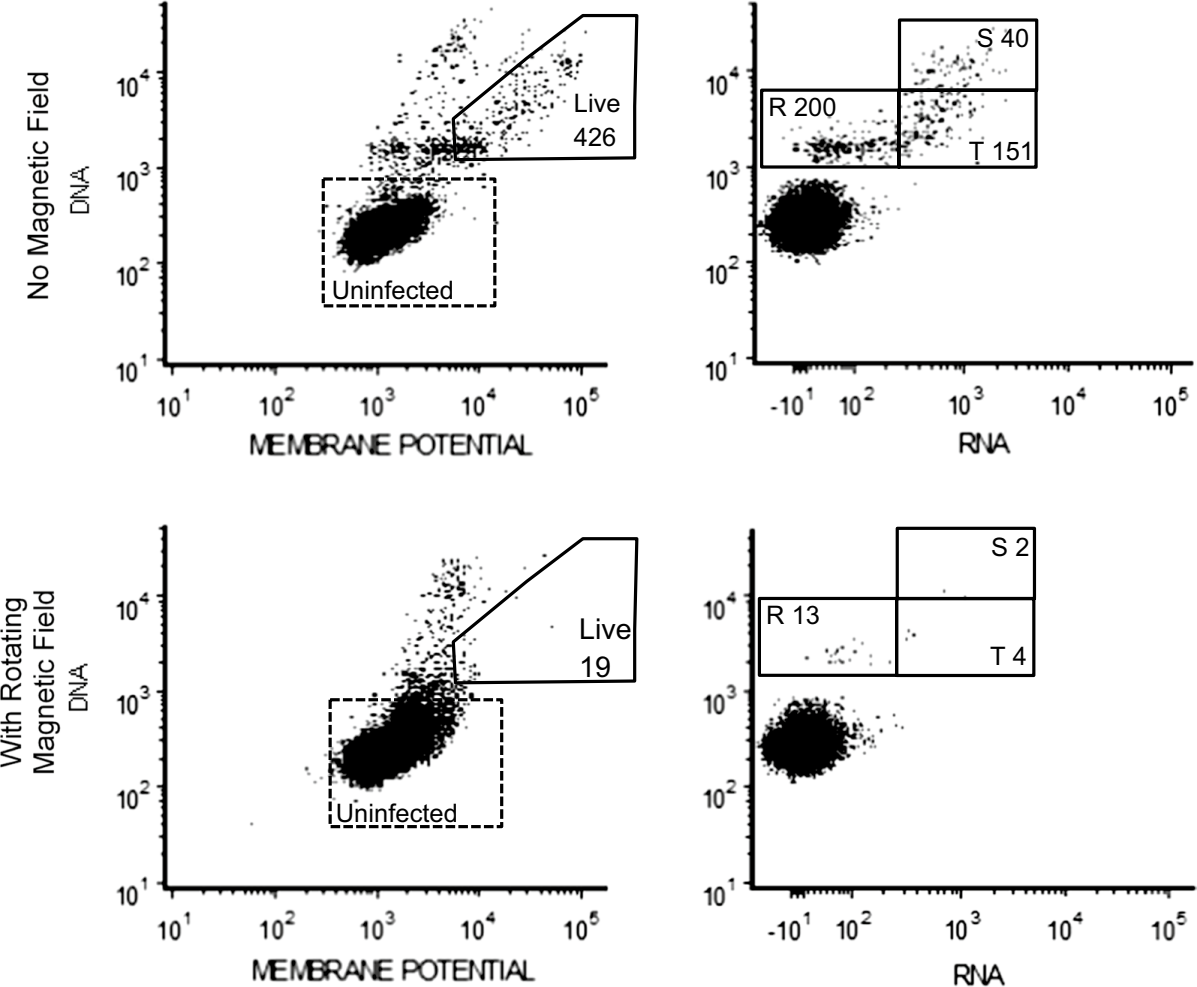
This graph is used to determine the number of infected RBCs with live parasites inside (membrane potential positive). An infected RBC will have a larger membrane potential and amount of DNA than an uninfected RBC shown in the left panels. The right hand panels shown the number of Rings (R), Trophozoites (T) and Schizonts (S) observed with 10 Hz (bottom panels) and without any (top panels) rotating magnetic fields. The uninfected cells are shown in the right-hand panels for reference

The viability and life stages of the parasites were determined using an LSRII flow cytometer with the BD Throughput Sampler Options (Becton–Dickinson, Franklin Lakes, NJ). From each microtube 60 µL of resuspended RBCs was stained with a 200 µL flow cytometry stain: 3 mL 1xPBS, 0.93 µL Hoechst 33342, 3.03 µL thiazole orange, 3.03 µL propidium iodide, and 3.01 µL DilC1-5. Hoechst 33342 is a DNA-specific fluorescent dye that determines if the cell is DNA positive or negative [17]. Likewise, thiazole orange is a fluorescent dye favoring RNA. Together, the two dyes can categorize the RBCs into infected or uninfected states, and, if infected, into the life stages: rings (DNA+/RNA−), trophozoites (DNA+/RNA+, < 4 nuclei), and schizonts (DNA+/RNA+, ≥ 4 nuclei) [18]. DilC1-5 was utilized to find the membrane potential of the cells to assess parasite viability. Propidium iodide was used to determine the viability of the RBC by staining cells with deteriorated cell membranes, but not to identify dead or dying parasites within live RBCs (Fig. 2) [17]. Flow was performed in duplicate as a check of cell counting.

To determine the effect of the magnetic field on the growth and death of the parasite the m-value was calculated,

1 $$m = \frac{{{{T_{live} } \mathord{\left/ {\vphantom {{T_{live} } {T_{dead} }}} \right. \kern-0pt} {T_{dead} }}}}{{{{C_{live} } \mathord{\left/ {\vphantom {{C_{live} } {C_{dead} }}} \right. \kern-0pt} {C_{dead} }}}}$$

where *T* is the number of live or dead infected RBCs in the culture exposed to the magnetic field, and *C* is the number of live or dead infected RBCs in the control culture. If *m* > 1 the test culture grew compared to the control culture, meaning the magnetic field aided the growth of the parasites. If *m* = 1, the test culture grew the same as the control and the magnetic field had no effect on the growth of the parasites. If *m* < 1, the test culture died compared to the control, meaning the magnetic field suppressed the growth of the parasite and actively killed them. With flow cytometry the death of the parasite, not only the growth inhibition can be taken into account, one of the main distinctions between our research and previous work [19].

### Microscopy

Standard light microscopy was used to observe parasitized RBCs blood smears as described previously [18, 20]. In brief, thin smears were prepared by spreading 5 µL of blood from cultures with a glass slide, fixed in 100% methanol, stained in 4% Giemsa (Sigma-Aldrich, St. Louis, MO), and examined by oil immersion LM (1000×).

### Theory and calculations

As discussed, haemozoin leads to a net magnetization associated with paramagnetism when exposed to a magnetic field. In particular, the iron atom at the centre of each haem has five unpaired electrons. According to Hund’s rule, energy is minimized when the total spin is maximized; therefore, the iron is in a spin *S* = 5/2 state. Since the haemozoin crystal is anisotropic, the magnetic susceptibility is a tensor. Therefore, with the application of a magnetic field, the induced magnetization is not parallel to the magnetic field, and the crystal will undergo a torque. An analysis of the susceptibility tensor shows that the long axis of the crystal will tend to align perpendicularly to the magnetic field [10].

To get an idea as to whether a field of 0.46 T is sufficient to cause rotation of a haemozoin crystal, the ratio of the magnetic rotational energy to the thermal energy can be calculated. The magnetic anisotropy energy is given by Butykai et al. [10].

$$U = \frac{1}{2}\frac{{B^{2} }}{{\mu_{0} }}\cos^{2} \theta (\chi_{xx} - \chi_{zz} )V$$

where *B* is the magnitude of the magnetic field, *χ*_*xx*_ and *χ*_*zz*_ are the tensor components of the volume susceptibility for the crystal long axis along the *z* direction, *θ* is the angle between the crystal long axis and the magnetic field vector, and *V* is the volume of a haemozoin crystal.

The susceptibility components are obtained by referring to Butykai et al. [10] At a temperature *T* = 295 K, the magnetizations are $M_{x} = 0.014{\mkern 1mu} \mu_{B} /{\text{Fe}}$ and $M_{z} = 0.012{\mkern 1mu} \mu_{B} /{\text{Fe}}$, where *μ*_*B*_ = 9.274 × 10^−24^ J/T is the Bohr magneton. By noting that there are 2 iron atoms per crystal unit cell and that the volume of the crystal unit cell is 1.416 × 10^−21^ cm^3^ [21], these units of Bohr magneton per iron atom are converted to the more familiar units of A/m by multiplying these quantities by the number of iron atoms per unit volume. The susceptibility components are obtained from *χ*_*xx*_ = *μ*_0_*M*_*x*_/*B* and *χ*_*zz*_ = *μ*_0_*M*_*z*_/*B* [10], giving in SI units *χ*_*xx*_ = 4.61 × 10^−4^ and *χ*_*zz*_ = 3.95 × 10^−4^. These values are comparable to the value of 3.2 × 10^−4^ given in Coronado et al. [21]. The ratio of magnetic to thermal energy may be written as

$$\frac{U}{kT} = \frac{{B^{2} }}{{2\mu_{0} }}(\chi_{xx} - \chi_{zz} )\frac{V}{kT}$$

Assuming crystal dimensions of 600 nm by 200 nm by 200 nm, a field magnitude of 0.46 T, and a temperature of 295 K, and noting that *μ*_0_ = 4*π* × 10^−7^ Tm/A the ratio of the magnetic to thermal energy is found to be 39. Since the magnetic energy is more than a magnitude greater than the thermal energy, the rotation of a haemozoin crystal is seen to be quite reasonable.

## Results

**Fig. 3:**
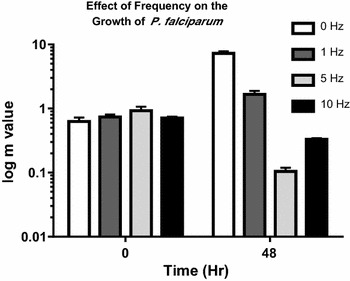
The m-value of a culture of all stages (rings, trophozoites, and schizonts) of *Plasmodium falciparum* culture shows the effect of variable frequency on growth. Each condition was tested in triplicate and standard error bars are shown for all samples

In order to clearly show the effect of the magnetic field the data are presented in two ways. First, the m-values for a culture of mixed stage parasites with an applied magnetic field at four different frequencies were investigated (Fig. 3). The static magnetic field clearly showed an increase in parasite growth compared to the control. The rotating magnets had a negative effect on parasite growth depending on the frequency. It appeared that the greater the frequency the smaller the parasite growth. After 48 h the m-value of the culture exposed to the 0 Hz or static field increased by 15-fold, the culture exposed to a 1 Hz field increased by threefold, the culture exposed to 5 Hz decreased by 8.9-fold, and the culture exposed to the 10 Hz field decreased by 2.15-fold (Fig. 3).

**Fig. 4:**
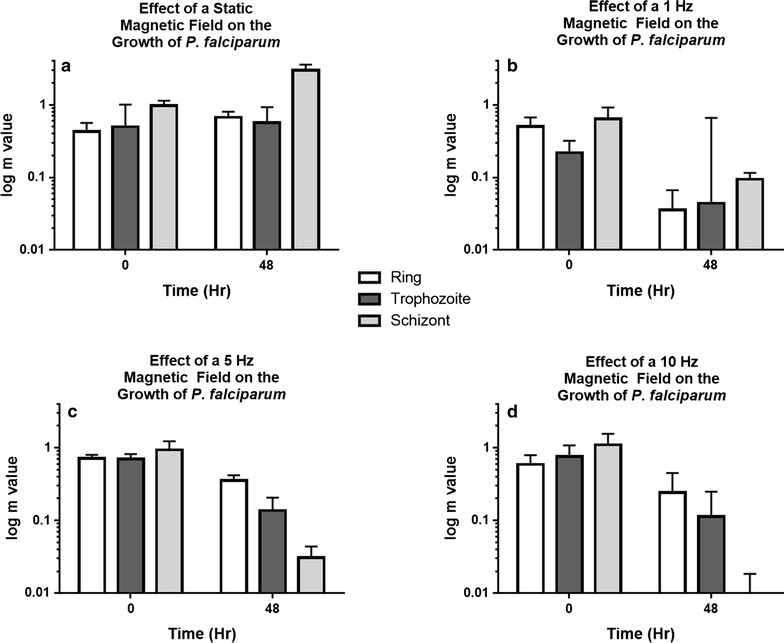
The m-value of each culture exhibiting the effect of variable frequency on the growth of the three different life stages of *Plasmodium falciparum*

Next, how the m-value changes depending on the initial life stage of the parasite (rings, trophozoites and schizonts) was explored. The error was determined by calculating the standard error for the flow cytometry data as propagated via the derivative method. Flow cytometry was performed on each condition in triplicate. The mechanism whereby the magnetic field was affecting culture growth or death can partially be elucidated by looking at the m-value of specific life stages (Fig. 4).

## Discussion

These results demonstrate that rotational frequency is proportional to parasite death. The increased growth observed in the presence of a static field may indicate that aligned crystals process waste faster allowing for improved growth rate, compared with the random motion in the absence of a static field at higher frequency. In the presence of a static field, the magnetic field is aligning (perpendicularly) with the haemozoin crystals, thereby reducing the effects of random thermal motion and parasitic digestion. In this case, the free haem molecules will more easily bind to the ends of the haemozoin crystal, reduce the energy expenditure of the parasite, and allow reduced energy utilization for replication. A similar mechanism could be used to explain the growth of the 1 Hz culture. The schizont culture was the only one that grew when exposed to a static magnetic field. Schizonts have fully formed haemozoin crystals, unlike trophozoites. This supports the hypothesis that the field helped align the haemozoin crystals, reducing the energy needed to process free haem and, therefore, aided the growth of the parasites. At 5 Hz the number of schizonts were suppressed by a factor of 30, while the trophozoites were suppressed by a factor of 5. At 10 Hz the schizonts were down by a factor of 120, while the trophozoites were down by a factor of 7. The increase in schizont death reveals that the higher rotating magnetic field is influencing the fully formed haemozoin crystals and not their formation. The decrease in m-value for the ring culture most likely occurs when they are cycling through the trophozoite and schizont stage; however, it is unclear why the ring culture decreases. It may be because the ring is forming haemozoin under the influence of the magnetic field, while the schizonts have fully formed haemozoin.

**Fig. 5:**
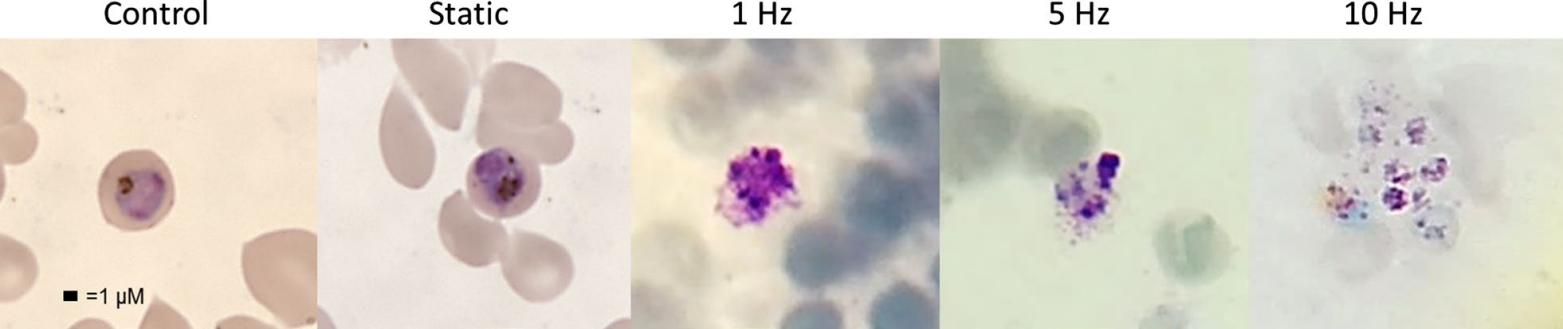
Representative images of trophozoites from control and each treatment demonstrates the overall damage to the parasites that results from the effects of the rotating magnetic field. It is important to note that parasites from the static magnetic field do not show an overall difference compared with control parasites. It is difficult to assess any specific changes to food vacuoles as a result of the rotating magnetic field because of the general destruction to the entire parasite

Two possible explanations for the death of the parasites at 5 and 10 Hz may be given. The first is that the magnetic field is disrupting the formation of haemozoin by manipulating the haemozoin crystal and causing an accumulation of toxic haem that is killing the parasite. The second is that the rotation of the haemozoin crystal due to the magnetic field is physically destroying the inside of the parasite as seen in Fig. 5.

## Conclusions

This study is the first to show the effect of a rotating magnetic field on the growth of *Plasmodium falciparum*, to construct a way of quantifying not only the growth of the parasites but also their death, and to show that a static field aids parasite growth. The rotating magnetic field appears to be affecting fully formed haemozoin crystals, not the formation of them.

In the future, the experiment will be repeated at 1 Hz in order to reduce the error in the results and to see if the parasites grow in a magnetic field for a longer time period, particularly the ring culture, as those parasites experience less mortality. In addition, an asymmetric rotating magnetic field could provide two mechanisms for disrupting haemozoin: the first is the rotation of the field and the second is the attractive force between the haemozoin and a gradient magnetic field. This does not occur in the current apparatus because the magnetic field is symmetric. Disruptive measures can be added, such as ferromagnetic particles and antimalarial drugs, to infected cultures to determine what effects they have on parasite growth with and without a rotating magnetic field.

In addition to changes in the nature of the magnetic field, it would be interesting to see the effect of the field on different strains of malaria that form different shapes. For example, *Plasmodium knowlesi* has diffuse haemozoin throughout the parasite instead of being constrained to the lipid body.

One can also consider the possible treatment applications for malaria patients. However, the magnetic field strength and frequency would first have to be optimized to cause the greatest malaria infected cell death in the shortest amount of time. Instead of applying a magnetic field to the whole body of the patient, a smaller apparatus could be constructed into which a patient’s extremity is placed. As the blood circulates throughout the body each parasite would be exposed to the magnetic field. Depending on the time needed to kill all parasites, patients could either receive one long exposure or a series of shorter exposures over a period of time. This new form of treatment would be beneficial as it would reduce the increasing number of new drug resistant parasites [22].

## References

[1] WHO. World malaria report, 2015. Geneva: World Health Organization; 2015.

[2] Elliott DA, McIntosh MT, Hosgood HD, Chen S, Zhang G, Baevova P (2008). Four distinct pathways of hemoglobin uptake in the malaria parasite *Plasmodium falciparum*. Proc Natl Acad Sci USA.

[3] Klonis N, Dilanian R, Hanssen E, Darmanin C, Streltsov V, Deed S (2010). Hematin-hematin self-association states involved in the formation and reactivity of the malaria parasite pigment, hemozoin. Biochemistry.

[4] Toh SQ, Glanfield A, Gobert GN, Jones MK (2010). Heme and blood-feeding parasites: friends or foes?. Parasit Vectors.

[5] Feagin JE, Wurscher MA, Ramon C, Lai HC. Magnetic fields and malaria. In: Biologic effects of light: proceedings of the biologic effects of light symposium. Hingham: Kluwer Academic Publishers; 1999.

[6] Casabianca LB, An D, Natarajan JK, Alumasa JN, Roepe PD, Wolf C (2008). Quinine and chloroquine differentially perturb heme monomer-dimer equilibrium. Inorg Chem.

[7] Uhlemann A, Staalsoe T, Klinkert M, Hviid L (2000). Analysis of *Plasmodium falciparum*-infected red blood cells. MACS & more.

[8] Moore LR, Fujioka H, Williams PS, Chalmers JJ, Grimberg B, Zimmerman PA (2006). Hemoglobin degradation in malaria-infected erythrocytes determined from live cell magnetophoresis. FASEB J.

[9] Griffiths D (1999). Introduction to electrodynamics.

[10] Butykai A, Orban A, Kocsis V, Szaller D, Bordacs S, Tatrai-Szekeres E (2013). Malaria pigment crystals as magnetic micro-rotors: key for high-sensitivity diagnosis. Sci Rep.

[11] Orban A, Butykai A, Molnar A, Prohle Z, Fulop G, Zelles T (2014). Evaluation of a novel magneto-optical method for the detection of malaria parasites. PLoS ONE.

[12] Orban A, Rebelo M, Molnar P, Albuquerque IS, Butykai A, Kezsmarki I (2016). Efficient monitoring of the blood-stage infection in a malaria rodent model by the rotating-crystal magneto-optical method. Sci Rep.

[13] Grimberg BT, Grimberg KO (2016). Hemozoin detection may provide an inexpensive, sensitive, 1-minute malaria test that could revolutionize malaria screening. Expert Rev Anti Infect Ther.

[14] Grimberg BT, Jaworska MM, Hough LB, Zimmerman PA, Phillips JG (2009). Addressing the malaria drug resistance challenge using flow cytometry to discover new antimalarials. Bioorg Med Chem Lett.

[15] Ljungström I, Perlmann H, Schlichtherle M, Scherf A, Wahlgren M (2004). Methods in Malaria Research.

[16] Newman DM, Heptinstall J, Matelon RJ, Savage L, Wears ML, Beddow J (2008). A magneto-optic route toward the in vivo diagnosis of malaria: preliminary results and preclinical trial data. Biophys J.

[17] Grimberg BT (2011). Methodology and application of flow cytometry for investigation of human malaria parasites. J Immunol Methods.

[18] Grimberg BT, Erickson JJ, Sramkoski RM, Jacobberger JW, Zimmerman PA (2008). Monitoring *Plasmodium falciparum* growth and development by UV flow cytometry using an optimized Hoechst-thiazole orange staining strategy. Cytometry A.

[19] Shapiro HM, Apte SH, Chojnowski GM, Hanscheid T, Rebelo M, Grimberg BT. Cytometry in malaria–a practical replacement for microscopy? Curr Protoc Cytom. 2013;11–20. 10.1002/0471142956.cy1120s65 23835802

[20] McNamara DT, Kasehagen LJ, Grimberg BT, Cole-Tobian J, Collins WE, Zimmerman PA (2006). Diagnosing infection levels of four human malaria parasite species by a polymerase chain reaction/ligase detection reaction fluorescent microsphere-based assay. Am J Trop Med Hyg.

[21] Coronado LM, Nadovich CT, Spadafora C (2014). Malarial hemozoin: from target to tool. Biochim Biophys Acta.

[22] Grimberg BT, Mehlotra RK (2011). Expanding the antimalarial drug arsenal-now, but how?. Pharmaceuticals (Basel).

